# Supplementary material for: Single-droplet surface-enhanced Raman scattering decodes the molecular determinants of liquid-liquid phase separation
Source: Nat Commun. 2022 Jul 28;13:4378. doi: 10.1038/s41467-022-32143-0 (PMC9334365; doi:10.1038/s41467-022-32143-0)
Supplement: Supplementary file 1 — Supplementary Information [file 41467_2022_32143_MOESM1_ESM.pdf]

# Supplementary Information

## Single-Droplet Surface-Enhanced Raman Scattering Decodes the Molecular Determinants of Liquid-Liquid Phase Separation

Anamika Avni,[1,2 §] Ashish Joshi,[1,3 §] Anuja Walimbe,[1,3] Swastik G. Pattanashetty,[1,3] Samrat Mukhopadhyay[1,2,3*]

[1]Centre for Protein Science, Design and Engineering, [2]Department of Chemical Sciences, [3]Department of Biological Sciences, Indian Institute of Science Education and Research (IISER) Mohali, Punjab, India.

[§]Contributed equally *Corresponding author. Email: mukhopadhyay@iisermohali.ac.in

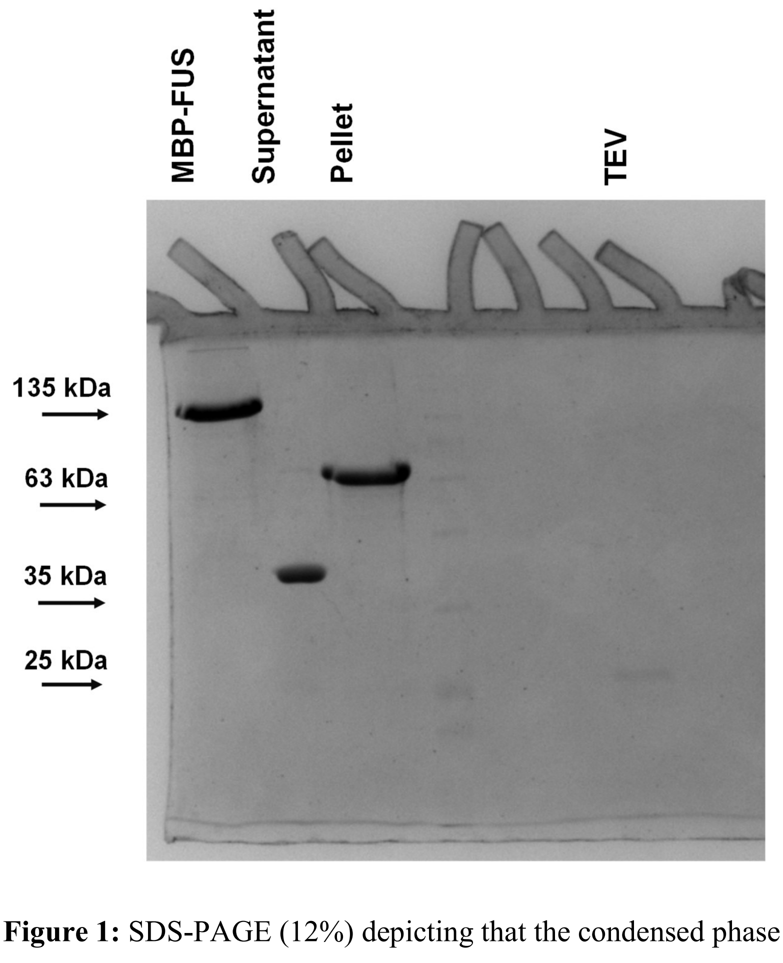


**Supplementary Figure 1:** SDS-PAGE (12%) depicting that the condensed phase is devoid of the maltose-binding protein (MBP) tag. The experiment was performed twice with similar observations.

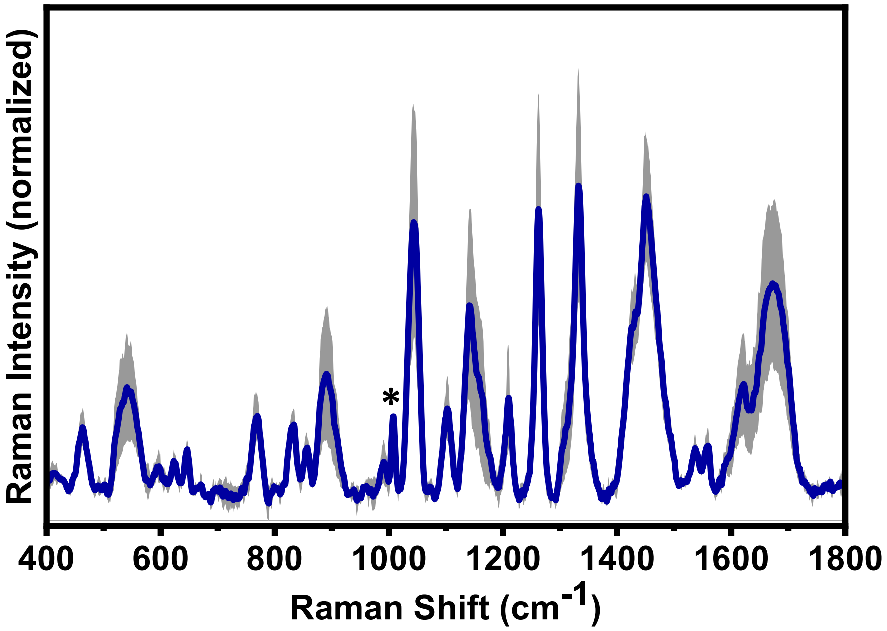


**Supplementary Figure 2:** Average single-droplet normal Raman spectra of FUS droplets (spectra recorded at 500 mW laser power, 100x objective, 10 accumulations; number of droplets, n = 3). Solid lines represent the mean, whereas shaded region represents the standard deviation. All spectra are normalized with respect to the phenylalanine ring breathing band at 1007 $cm^{-1}$ marked by an asterisk. See Methods for details of data acquisition, processing, and analysis.

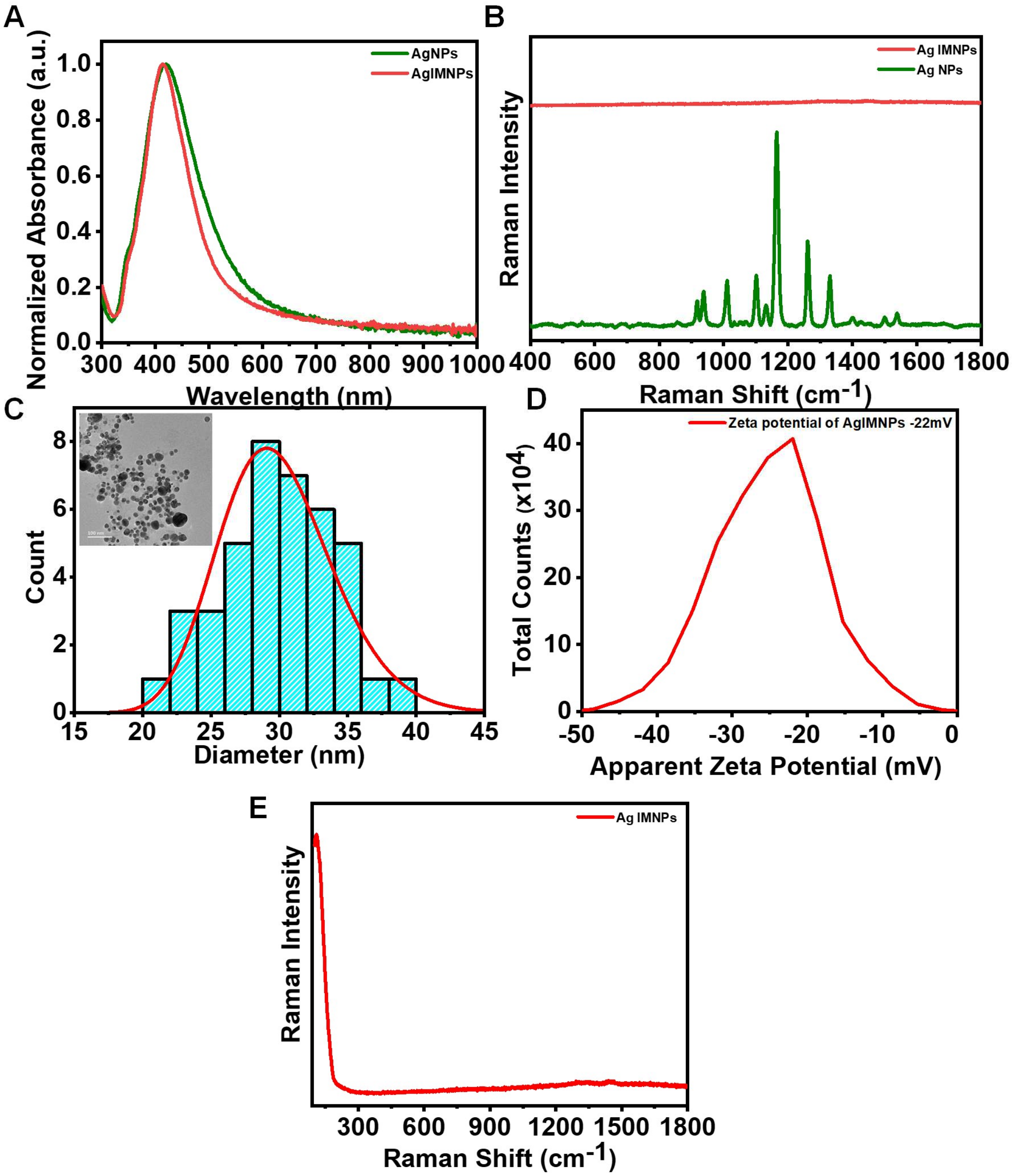


**Supplementary Figure 3: Preparation and characterization of iodide-modified silver nanoparticles (Ag IMNPs).** (A) UV-visible absorption spectra for silver nanoparticles (Ag NPs) (olive) and iodide-modified silver nanoparticles (Ag IMNPs) (red). (B) Raman spectra for silver nanoparticles (Ag NPs) (olive) and Ag IMNPs (red). (C) Histogram for nanoparticles size distribution derived from the TEM analysis. Size analysis performed by considering sizes of 40 different nanoparticles. Inset shows TEM image of Ag IMNPs. Scale bar: 100 nm. (D) Zeta-potential for Ag IMNPs. Plotted here is the mean of three different measurements. (E) SERS spectrum of Ag IMNPs corresponding to the Ag-I bond at 110 $cm^{-1}$.

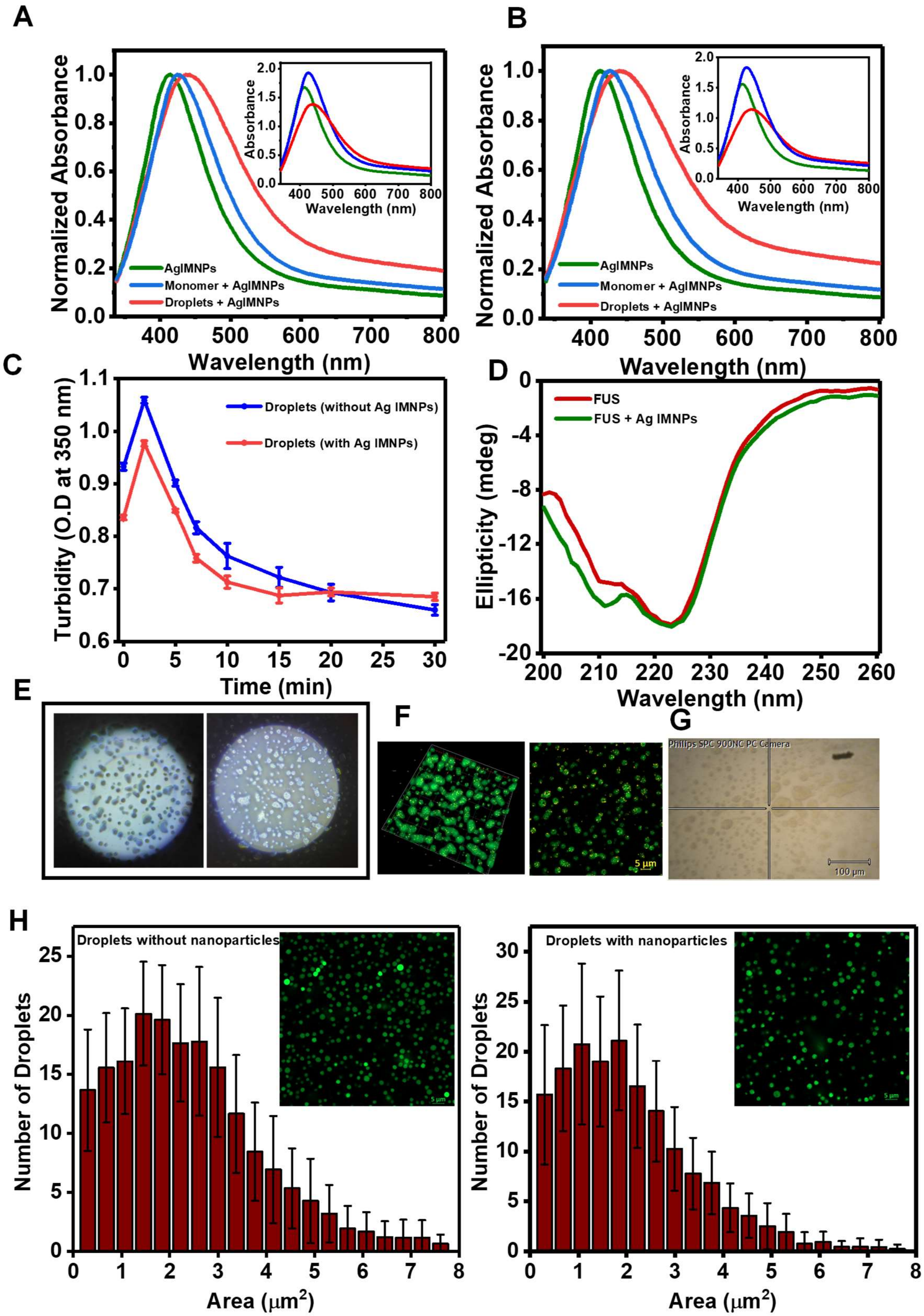


**Supplementary Figure 4: Interaction of FUS with iodide-modified silver nanoparticles (Ag IMNPs).** UV-Visible absorption spectra of Ag IMNPs in phosphate buffer (olive), monomeric FUS in the presence of Ag IMNPs (blue), FUS droplets in the presence of Ag IMNPs (red) at 10 minutes (A) and 30 minutes (B). Inset shows unnormalized UV-visible

absorption spectra. (C) Turbidity plot of FUS droplets in the absence (blue) and presence (red) of Ag IMNPs (mean ± s.d. for n = 4 independent experiments). (D) Far-UV CD spectrum of monomeric FUS and FUS in the presence of Ag IMNPs (5uM protein, 0.025 nM Ag IMNPs, 20 mM sodium phosphate, pH 7.4). (E) Eye-piece image for FUS droplets without and with Ag IMNPs. (F) 3-dimentional confocal image showing the presence of Ag IMNPs within FUS droplets. Confocal microscopy image of fluorescein-5-maleimide-labeled FUS and Ag IMNPs indicating encapsulation of Ag IMNPs within the droplets. (G) Encapsulation of Ag IMNPs as seen through a Raman microscope using 50x objective lens. All the imaging experiments were performed atleast thrice with similar observations. (H) Size analysis of confocal images from 2D projection area showing the area distribution plot obtained from the image analysis using ImageJ. Estimated mean diameter of FUS droplets in the absence of Ag IMNPs was ~ 1.7 µm and in the presence of Ag IMNPs was 1.8 µm. For the analysis, the number of independent images used was 28 (without nanoparticles) and 29 (with nanoparticles). The data represents mean ± s.d. for n = 28 (without nanoparticles) and n = 29 (with nanoparticles). Representative confocal images are also shown in insets (scale bar: 5 µm). Similar size distributions suggested that nanoparticles do not significantly alter the droplet size.

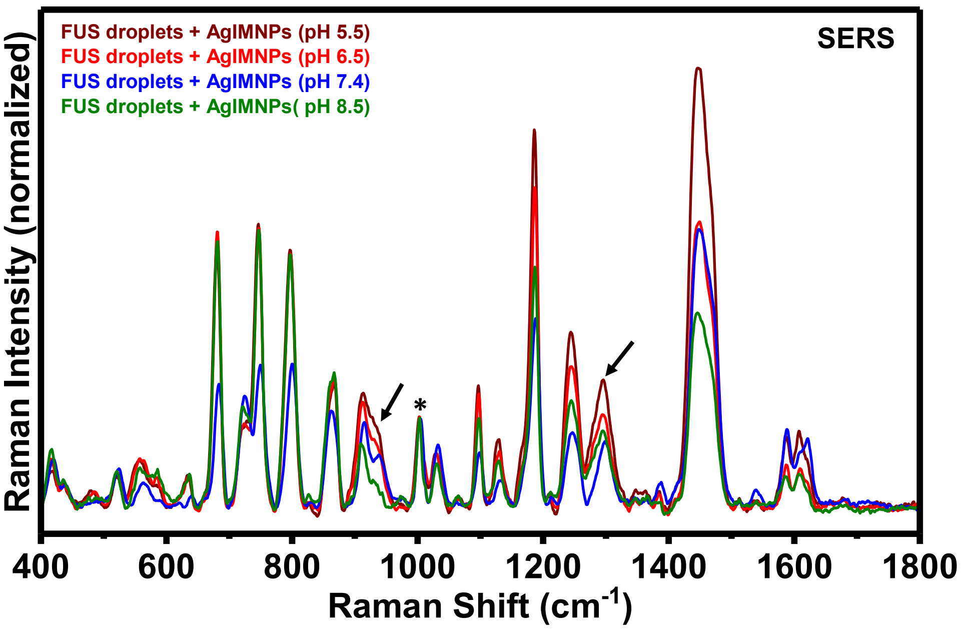


**Supplementary Figure 5: Raman spectra of FUS droplets at different pH values.** (A) Average single-droplet SERS spectra from individual FUS droplets encapsulating Ag IMNPs at different pH values (spectra recorded at 5 mW laser power, 50x objective, 1 accumulation; number of droplets, n = 7). See Methods for experimental details and Supplementary Table 3 for all the band positions. All spectra are normalized with respect to the phenylalanine ring breathing band at 1003 $cm^{-1}$ marked by an asterisk. See Methods for details of data acquisition, processing, and analysis. Arrows at 940 $cm^{-1}$ and 1296 $cm^{-1}$ denotes greater α-helicity within FUS droplets as we go from pH 8.5 to 5.5. Peak at 940 $cm^{-1}$ denotes backbone C-C stretch of α-helices, and peak at 1296 $cm^{-1}$ represents Amide III band corresponding to α-helical structures.

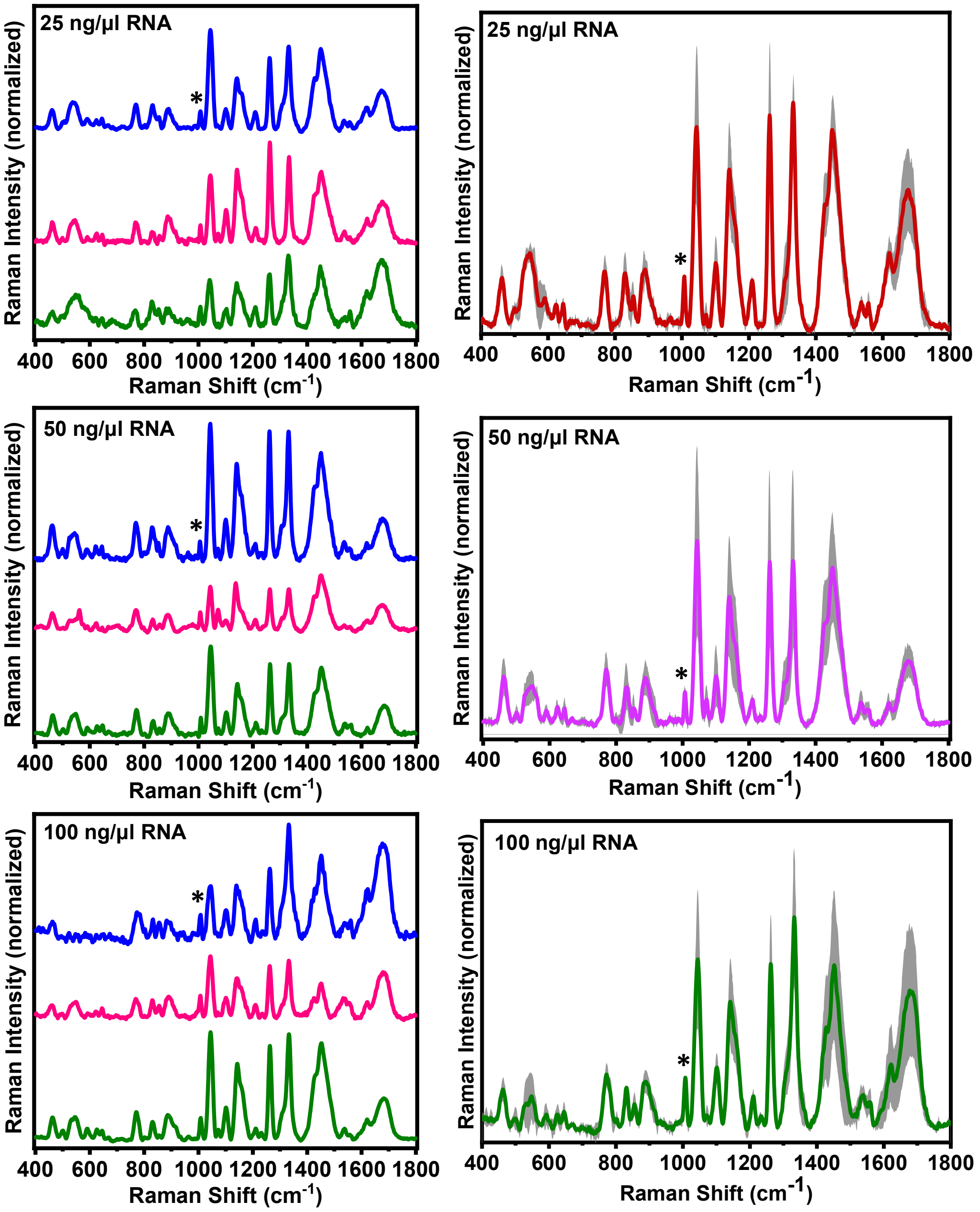


**Supplementary Figure 6: Single-droplet normal Raman spectra of FUS in the presence of RNA.** Stacked single-droplet normal Raman spectra of FUS droplets at different concentrations of RNA (25 ng/μL, 50 ng/μL, and 100 ng/μL) (spectra recorded at 500 mW laser power with a 100x objective, 10 accumulations and 10 sec exposure time; number of droplets, n = 3). Spectra on the right represent mean and the standard deviations. Solid lines represent the mean, whereas shaded region represents the standard deviation (n = 3).

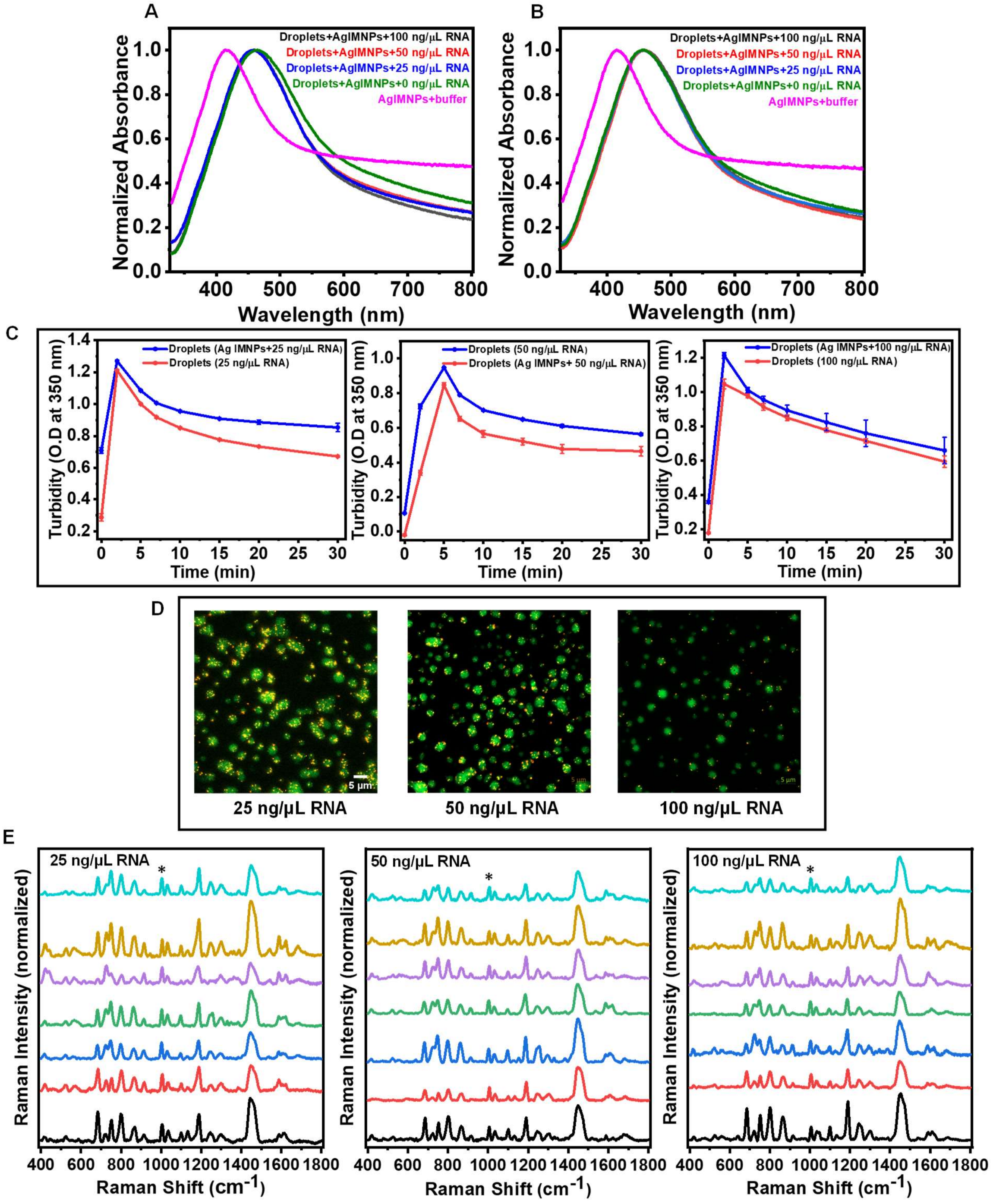


**Supplementary Figure 7: FUS droplets in the presence of Ag IMNPs and RNA.** (A,B) UV-Visible absorption spectra of Ag IMNPs in phosphate buffer (magenta) and FUS droplets at different concentrations of RNA (0 ng/ µL, 25 ng/µL, 50 ng/µL, and 100 ng/µL) in the presence of Ag IMNPs at 10 minutes and 30 minutes. (C) Solution turbidity plot of FUS droplets for different concentration of RNA (25 ng/µL, 50ng/µL, and 100 ng/µL) in the absence and

presence of Ag IMNPs (mean ± s.d. for n = 4 independent experiments; 25 ng/μL and 100 ng/μL RNA, n = 3; 50 ng/μL RNA). (D) Confocal microscopy images of FUS droplets for different concentrations of RNA (25 ng/μL, 50 ng/μL, and 100 ng/μL) in the presence of Ag IMNPs. The experiments were performed thrice with similar observations. (E) Stacked SERS spectra of FUS droplets at different concentrations of RNA (25 ng/μL, 50 ng/μL, and 100 ng/μL) (spectra recorded at 5 mW laser power with a 50x objective; 1 accumulation and 10 sec exposure time; number of droplets, n = 7).

**Supplementary Table 1.** Common vibrational bands of protein Raman spectra.[1-4]

| | |
|---|---|
| **Amide Bands**<br><br>Amide bands represent motion of atoms of the peptide backbone and determines the secondary structure of the proteins.<br><br>**Amide I** (primarily a carbonyl stretching mode)<br><br>**Amide III** (combines in-plane N-H bending and C-N stretching motions) | ○ α-helix: ~1630-1655 $cm^{-1}$<br>○ β-sheet: ~1669-1675 $cm^{-1}$<br>○ Non-regular/disordered structures: ~1655-1669 $cm^{-1}$ and 1675-1690 $cm^{-1}$<br><br>○ α-helix: ~1280-1320 $cm^{-1}$<br>○ β-sheet: ~1220-1240 $cm^{-1}$<br>○ Non-regular/disordered structures: ~1250-1270 $cm^{-1}$ |
| **Disulfide stretch (S-S) and carbon-sulfur (C-S) bands**<br><br>Raman bands due to disulfide bonds appear in 500-550 $cm^{-1}$ region.<br>Different conformations of atoms around S-S bonds appear at different wavenumbers.<br><br>C-S bands of cysteine and methionine residues appear depending on sidechains and surrounding environment. | ○ Gauche-gauche-gauche (g-g-g): ~515 $cm^{-1}$<br>○ Gauche-gauche-trans (g-g-t): ~525 $cm^{-1}$<br>○ Trans-gauche-trans (t-g-t): ~540 $cm^{-1}$<br><br>○ 630-760 $cm^{-1}$ |

| | |
|---|---|
| **Aromatic amino acids**<br><br>**Phenylalanine**<br>• Intense band due to ring breathing vibrations and is often used for the normalization of the Raman spectra of proteins as this band is independent of conformational changes of the proteins.<br><br>**Tryptophan**<br>• Indole ring-breathing vibrations<br><br>• Indole N-H bending vibrations and is sensitive to indole N-H hydrogen bond donation<br><br>• Fermi doublet at 1360 $cm^{-1}$ and 1340 $cm^{-1}$<br><br>**Tyrosine**<br>• Fermi doublet at 850 $cm^{-1}$ and 830 $cm^{-1}$ observed due to Fermi resonance between the ring-breathing vibrations and overtone of an out-of-plane ring-bending vibration of the phenolic ring of tyrosine.<br><br>• Ring stretching mode<br><br>• C-O stretching mode<br><br>• C-C stretching mode<br><br>• O-H bend + C-H bend | ○ 1000 $cm^{-1}$<br><br>○ ~ 760 $cm^{-1}$<br>○ Marker band for cation-π/CH-π interactions<br>○ Band intensity increases with decreasing hydrophobicity.<br><br>○ ~ 880 $cm^{-1}$<br>○ Blue shift indicates indolyl moiety is located in highly hydrophobic environment.<br>○ Red shift indicates strong H-bonding representing strength of N-H bond of the indole ring with surrounding solvent molecules.<br><br>○ $I_{1360}/I_{1340}$ increases with increasing hydrophobicity of indole ring environment.<br><br>○ $I_{850}/I_{830}$ is an indicator of solvent-mediated hydrogen bonding propensity of the phenolic (-OH) group<br><br>○ 1617 $cm^{-1}$ & 1600 $cm^{-1}$<br><br>○ 1263 $cm^{-1}$<br><br>○ 1210 $cm^{-1}$<br><br>○ 1180 $cm^{-1}$ |
| **Backbone $CH_2/CH_3$ deformations** | ~ 1440-1470 $cm^{-1}$ |
| **Backbone $^{\alpha}$C-H bending vibrations** | ~ 1390 $cm^{-1}$ |

**Supplementary Table 2.** Percentage analysis of δ(NH)-guanidinium moiety of arginine residues and $CH_2/CH_3$ deformation modes obtained after deconvolution of the region 1420-1490 $cm^{-1}$ for the single-droplet SERS spectra for various concentrations of RNA (0, 50, and 100 ng/μL).

| FUS droplets with Ag IMNPs with RNA | **δNH; Guanidinium moiety (1447 $cm^{-1}$) (in %)** | **$CH_2/CH_3$ deformations (1468 $cm^{-1}$) (in %)** |
|---|---|---|
| 0 ng/μL RNA | 71 ± 13 | 44 ± 13 |
| 50 ng/μL RNA | 68 ± 8 | 27 ± 8 |
| 100 ng/μL RNA | 60 ± 10 | 38 ± 10 |

**Supplementary Table 3.** Raman shift values and tentative band assignments of normal Raman and SERS spectra of full-length FUS droplets.[1-4]

| **Single-droplet Normal Raman** | **Single-droplet SERS** | **Peak assignments**[§] |
|---|---|---|
| 1673 (s) | - | Amide I (β-sheet) |
| 1621 (s) | 1621 (s) | Tyr (R stretch) |
| - | 1588 (s) | Phe, Trp, His |
| 1557 (m) | - | Trp |
| 1537 (w) | 1538 (w) | Trp, Amide II |
| - | 1447 (s) | δ(NH)-guanidinium moiety |
| 1451 (s) | - | $\delta(CH_2/CH_3)$ |
| - | 1388 (w) | Asp, Glu $\nu_{sy}[COO^-]$ |
| 1332 (s) | - | Trp, δ(CαH) |
| - | 1298 (s) | Amide III (α-helix) |
| 1262 (s) | - | Amide III (nonregular/turns) |
| - | 1246 (s) | Amide III (β-sheet) |
| 1209 (s) | 1213 (w) | Tyr [ν(C-C)] |
| - | 1188 (s) | Tyr, Phe, ν(C-N) |
| 1140 (s) | 1132 (m) | $\nu_{as}$(CαCN) |
| 1101 (s) | 1098 (s) | ν(C-C), ν(C-O), ν (C-N) |
| 1042 (s) | 1032 (s) | Phe [δ(R(CH))] |

| | | |
|---|---|---|
| 1006 (s) | 1003 (s) | Phe R breathing |
| 958 (w) | - | ν(N-Cα-C) skeletal |
| - | 938 (s) | Backbone skeletal (α-helix) |
| - | 915 (s) | ν($COO^-$), C-C stretch of Pro ring |
| 890 (s) | - | Trp (N-H bend) |
| 857 (s) | 862 (s) | Tyr Fermi doublet |
| 830 (s) | - | Tyr Fermi doublet |
| 798 (w) | 800 (s) | ν(C-H), δ(N-H), Met [$\nu_{as}$(C-S-C)] |
| 767 (s) | 749 (s) | Trp [δ($R_{breathing}$)] |
| - | 724 (s) | Met [ν(C-S)] |
| - | 683 (s) | Met [ν(C-S)], δ(C-H) |
| 646 (m) | 640 (w) | Tyr [γ(C-C)] |
| 623 (w) | - | Tyr, ν(C-S) |
| 597 (m) | - | δ($COO^-$) |
| - | 562 (m) | ν(S-S) |
| 539 (s) | 522 (m) | δ(skeletal), δ(N-H), ν(S-S) |
| 461 (s) | - | ν(C-S) |
| - | 419 (m) | Trp |

[§]δ, bending; ν, stretching; R, benzene ring; as, asymmetric; sy, symmetric; w, weak; m, medium; s, strong

**References**


1. Rygula, A., Majzner, K., Marzec, K. M., Kaczor, A., Pilarczyk, M., Baranska, M. Raman Spectroscopy of Proteins: A Review. *J. Raman Spectrosc.* **44,** 1061-1076 (2013).
2. Tuma, R. Raman Spectroscopy of Proteins: From Peptides to Large Assemblies. *J. Raman Spectrosc*. **36,** 307-319 (2005).
3. Singh, S., Agarwal, A., Avni., Mukhopadhyay, S. Ultrasensitive Characterization of the Prion Protein by Surface-Enhanced Raman Scattering: Selective Enhancement via Electrostatic Tethering of the Intrinsically Disordered Domain with Functionalized Silver Nanoparticles. *J. Phys. Chem. Lett.* **12**, 3187-3194 (2021).
4. Szekeres, G. P., Kneipp, J. Probing of Proteins in Gold Nanoparticle Agglomerates. *Front. Chem*. **7,** 30, (2019).
