# Supplementary material for: Single-droplet surface-enhanced Raman scattering decodes the molecular determinants of liquid-liquid phase separation
Source: Nat Commun. 2022 Jul 28;13:4378. doi: 10.1038/s41467-022-32143-0 (PMC9334365; doi:10.1038/s41467-022-32143-0)
Supplement: Supplementary file 3 — Description of additional supplementary files [file 41467_2022_32143_MOESM3_ESM.pdf]

Description of Additional Supplementary Files

Supplementary Movie 1 : Multiple Z stack images taken at different focal distances showing iodide-modified silver nanoparticles encapsulated within the fluorescently labeled FUS droplets.
